# Triple motif proteins 19 and 38 correlated with treatment responses and HBsAg clearance in HBeAg-negative chronic hepatitis B patients during peg-IFN-α therapy

**DOI:** 10.1186/s12985-023-02119-7

**Published:** 2023-07-20

**Authors:** Haiying Luo, Guili Tan, Xiaoxia Hu, Yadi Li, Dingjia Lei, Yueying Zeng, Bo Qin

**Affiliations:** 1 Department of Infectious Diseases, Chongqing Key Laboratory of Infectious Diseases and Parasitic Diseases, the First Affiliated Hospital of Chongqing Medical University, No. 1 Youyi Road, Yuzhong District, Chongqing, 400016 China; 2 Central Laboratory, the First Affiliated Hospital of Chongqing Medical University, Chongqing, China

**Keywords:** Hepatitis B virus, Chronic carriers, TRIM19, TRIM38, peg-IFN-α, Virological response

## Abstract

**Objective:**

To investigate whether the expression of triple motif protein 19/38 (TRIM19/38) mRNA in peripheral blood mononuclear cells (PBMCs) of HBeAg-negative chronic hepatitis B virus (HBV) carriers is associated with the response to pegylated interferon alpha (peg-IFN-α) treatment and HBsAg clearance.

**Methods:**

In this prospective study, HBeAg-negative chronic HBV carriers treated with peg-IFN-α completed 48 weeks of follow-up. After treatment with peg-IFN-α, the patients were divided into responders (R group) and nonresponders (NR group) according to the changes in HBV DNA and HBsAg levels at week 48 of treatment. According to whether serum HBsAg loss or seroconversion occurred, the patients were divided into a serological response group (SR group) and a nonserological response group (NSR group). The level of TRIM19/38 mRNA in PBMCs was detected by real-time fluorescence quantitative PCR. The diagnostic performance of TRIM19/38 was analysed by calculating the receiver operating characteristic (ROC) curve and area under the ROC curve (AUC).

**Results:**

43 HBeAg-negative chronic HBV carriers, 35 untreated CHB patients and 19 healthy controls were enrolled in this study. We found that TRIM19/38 mRNA levels were significantly lower in untreated CHB patients than in healthy controls. In HBeAg-negative chronic HBV carriers who underwent prospective follow-up, TRIM19/38 mRNA levels were negatively correlated with HBV DNA and ALT at baseline. Among the patients treated with peg-IFN-α, 16 patients achieved a treatment response (R group) and 27 patients did not achieve a treatment response (NR group). Compared with baseline, HBsAg levels in the R group decreased significantly at 12 and 24 weeks of treatment; at the early stage of peg-IFN-α treatment, the dynamic changes in TRIM19/38 mRNA levels in the R and NR groups were different, and the TRIM19/38 mRNA levels in the R group were significantly higher than those in the NR group, especially at 24 weeks of treatment. ROC curve analysis showed that the changes in mRNA levels of TRIM19 and TRIM38 predicted the treatment response, with AUCs of 0.694 and 0.757, respectively. Among the patients treated with peg-IFN-α, 11 patients achieved a serological response (SR group) and 32 patients did not achieve a serological response (NSR group). Compared with baseline, HBsAg levels in the SR group decreased significantly at 12 and 24 weeks of treatment; TRIM19/38 mRNA levels were significantly higher in the SR group than in the NSR group at week 24.

**Conclusion:**

The higher level of TRIM19/38 mRNA in PBMCs of HBeAg-negative chronic HBV carriers may be related to the early treatment effect of peg-IFN-α and HBsAg clearance. TRIM19 and TRIM38 have clinical significance in predicting virological response and guiding treatment regimens.

## Introduction

Hepatitis B virus (HBV) infection remains a serious global health problem [1]. According to the latest report of the World Health Organization, there are approximately 296 million chronic HBV infections in the world [2]. Chronic HBV infection increases the development of advanced liver diseases such as cirrhosis, hepatic decompensation, and hepatocellular carcinoma (HCC) [3, 4]. Therefore, controlling the development of chronic HBV infection and eliminating HBV has become a hot issue in recent years.

It has been reported that approximately 10% of patients in the immune tolerant phase spontaneously transition to hepatitis B e antigen (HBeAg)-negative chronic HBV carriers each year [5]. Previous studies have suggested that such patients with chronic hepatitis B (CHB) have mild liver damage and are in a relatively good stage, so the guidelines for the prevention and treatment of CHB have not recommended active antiviral therapy for them [6, 7]. However, recent studies have reported that such patients may recover or progress to other severe stages of chronic hepatitis and even have the risk of developing end-stage liver disease [8, 9]. Therefore, there is a need for effective treatment and prediction of efficacy in HBeAg-negative chronic HBV carriers. There are currently two clinical treatment options for chronic HBV infection: nucleoside analogues (NAs) and pegylated interferon alpha (peg-IFN-α) [6, 8, 9]. Although NAs can play a role in the inhibition of HBV, they require long-term treatment. In contrast, peg-IFN-α can exert sustained HBV suppression in a limited course of treatment and can clear hepatitis B surface antigen (HBsAg) to achieve a “clinical cure” [10]. However, the use of peg-IFN-α has been hindered by its side effects. Therefore, it is critical to explore novel biomarkers for predicting the response to peg-IFN-α therapy.

Triple motif proteins (TRIMs) are a family of proteins that play an important role in antiviral innate immunity [11]. In recent years, an increasing number of studies have shown that members of the triple motif protein family can effectively play a role in the inhibition of HBV and are related to the treatment of interferon [12]. As a member of the triple motif protein family, TRIM38 is associated with viral infection and the interferon response [13, 14]. In our previous study, we found that TRIM38 inhibited HBV replication and expression and could be induced by peg-IFN-α [15]. TRIM19, also known as PML (promyelocytic leukaemia protein), has long been found to be involved in the defence against various viruses [16, 17]. TRIM19 has been shown to indirectly inhibit a variety of DNA and RNA viruses by regulating the interferon response [18]. Recent studies have also suggested that TRIM19 may inhibit HBV by interacting with FoxO4 [19]. However, the dynamic changes in TRIM19 and TRIM38 during peg-IFN-α treatment and their correlation with treatment efficacy and HBsAg clearance have not been reported.

The aim of this study was to determine the relationship between TRIM19/38 mRNA levels in peripheral blood mononuclear cells (PBMCs) and the response to peg-IFN-α treatment and HBsAg clearance in HBeAg-negative chronic HBV carriers. Our study may help to evaluate the predictive value of TRIM19/38 mRNA levels in PBMCs of HBeAg-negative chronic HBV carriers for the efficacy of peg-IFN-α treatment and HBsAg clearance and provide help for the individualized treatment of peg-IFN-α.

## Materials and methods

### Patient population

This was a prospective observational cohort study in patients with CHB. We enrolled 43 HBeAg-negative chronic HBV carriers aged 18–65 years in the outpatient Department of Infectious Diseases of the First Affiliated Hospital of Chongqing Medical University from January 2021 to January 2022. The clinical diagnosis of HBeAg-negative chronic HBV carriers is based on the 2019 American Association for the Study of Liver Diseases (AASLD) consensus statement on managing chronic hepatitis B [7]. Enrolled patients met the following criteria: HBsAg positive, HBeAg negative, HBeAb positive, HBcAb positive, low HBV DNA load (< 2000 IU/ml), normal liver function, and no or only mild liver inflammation/fibrosis. Patients were excluded if they met any of the following criteria: (1) coinfected with other hepatitis viruses, such as hepatitis C virus (HCV) and hepatitis D virus (HDV); (2) coinfected with other viruses, such as human immunodeficiency virus (HIV) and EB virus (EBV); (3) autoimmune liver diseases; (4) severe liver damage or other liver diseases, such as alcoholic liver disease and fatty liver; (5) other malignant diseases; (6) contraindication for IFN. In addition, 32 untreated CHB patients and 19 healthy controls were included in the study. Written informed consent was obtained from all study participants. Our study was approved by the ethics committee of the First Affiliated Hospital of Chongqing Medical University (Refer-ence number: 3-2022).

HBeAg-negative chronic HBV carriers treated with peg-IFN-α were tested at baseline and every 12 weeks thereafter, including HBsAg levels, HBV DNA, liver function, and routine blood tests. Peripheral blood samples were obtained at baseline and after 12 and 24 weeks. Peripheral blood mononuclear cells (PBMCs) were isolated and the mRNA levels of TRIM19 and TRIM38 in PBMCs were measured. Untreated CHB patients and healthy controls were tested at baseline as described above.

We divided HBeAg-negative chronic HBV carriers into two groups based on the level of change in HBV DNA and HBsAg at week 48 of peg-IFN-α treatment. HBV DNA decreased by > 2 log10 IU/ml or HBsAg decreased rapidly during treatment (HBsAg decreased by > 1 log10 IU/ml or HBsAg clearance) after receiving peg-IFN-α for 48 weeks was defined as responders (R group); the remainder of the patients were defined as nonresponders (NR group). Serological response (SR group) was defined as serum HBsAg loss or HBsAg seroconversion during peg-IFN-α therapy. Instead, it was defined as a nonserological response (NSR group).

### Serological and virological assays

HBV DNA load was detected by real-time fluorescence quantitative PCR with a lower limit of quantification of 20 IU/ml (Roche, Cobas TM48, Shanghai). Serum HBsAg/HBsAb and HBeAg/HBeAb levels were detected by Abbott Architect i2000 Detection Reagent (Abbott Architect i2000). Liver function was detected by an automatic biochemical analysis detector (Roche, Cobas, Shanghai). Routine blood measurements were detected by automatic blood analyser (Mairui, BC-6600, Shanghai).

### RNA extraction and quantitative real-time PCR (qRT-PCR)

We determined the mRNA levels of TRIM38 and TRIM19 in PBMCs of CHB patients by two-step reverse transcription-polymerase chain reaction quantitative analysis. Total RNA was extracted from PBMCs using TRIzol reagent, and RNA was subsequently converted to first-strand cDNA using a complementary DNA (cDNA) synthesis kit. Then, cDNA was subjected to quantitative real-time PCR using a Bio-Rad fluorescence quantitative PCR instrument CFX96 (USA). The conditions are as follows: Predenaturation: 95℃ 30 s →PCR: (95℃ 5 s, 60℃ 30 s)×40 cycles→Melting solution: 95℃ 5 s, 60℃ 1 min, 95℃→Cooling: 50℃ 30 s→Read the board. The levels of target genes were determined based on the measure of relative quantification using β-actin as the reference gene. The primers are listed in Table 1.

**Table 1:** sequences of primer used in this study

primers	sequence(5’-3’)
β-actin RP	5’-CCTGGCACCCAGCACAAT-3’
β-actin FP	5’-GCCGATCCACACGGAGTA-3’
TRIM19 RP	5’-GGAACTTGCTTTCCCGCTTC-3’
TRIM19 FP	5’-CGGAAGACTCAGATGCCGAA-3’
TRIM38 RP	5’-GGCATACGTCTTCAACAAGAGC-3’
TRIM38 FP	5’-ACACGGAGAGCAGTTCCAC-3’

### Statistical analysis

Statistical analysis and mapping were performed using SPSS 20.0 and GraphPad Prism 8.0.

The results are presented as the means ± SDs unless stated otherwise. Independent sample t test were used to analyse normally distributed data, and Mann-Whitney nonparametric U test were used to analyse nonnormally distributed data. Spearman’s correlation coefficient was used to evaluate the correlation between baseline parameters. To assess the change in TRIM19/38 level, mRNA levels were first transformed with base 2 log to obtain a fold change in TRIM19/38(FC-TRIM19/38) FC = 2^(log2 ^(TRIM19/38week 24)^ ‐ log2 ^(TRIM19/38week 0)^) value was calculated for each patient and used for statistical analysis. The diagnostic performance of TRIM19/38 was analysed by calculating the receiver operating characteristic (ROC) curve and area under the ROC curve (AUC). The sensitivity, specificity, positive predictive value and negative predictive value were calculated at the optimal cut-off value. All statistical analyses were based on two-tailed hypothesis tests, and P < 0.05 was considered statistically significant.

## Results

### Baseline clinical characteristics of all subjects and serological response to peg-IFN-α therapy in HBeAg-negative CHB patients

Our study cohort consisted of 19 healthy controls (HCs, 11 males and 8 females), 32 untreated CHB patients (16 males and 16 females), and 43 HBeAg-negative CHB patients treated with peg-IFN-α (27 males and 16 females). The baseline characteristics of each group are shown in Table 2. As shown in Table 2, there were no significant differences in sex, age, ALT, AST, WBC and HBsAg in the groups (P < 0.05).

A total of 43 HBeAg-negative CHB patients were given 48 weeks of peg-IFN-α. CHB patients treated with peg-IFN-α experienced different periods of time to achieve serological responses. As shown in Table 3, one case achieved HBsAg clearance at 12 weeks after peg-IFN-α therapy, and the HBsAg clearance rates at 24, 36 and 48 weeks were 9.30% (4/43), 11.63% (5/43), and 25.58% (11/43), respectively. There was no HBsAg seroconversion at 12 weeks after peg-IFN-α therapy, and the HBsAg seroconversion rates at 24, 36 and 48 weeks were 6.98% (3/43), 9.30% (4/43), and 13.95% (6/43), respectively.

**Table 2:** Baseline characteristics in the groups

Characteristics	HC	Untreated CHB	CHB treated with peg-IFN-α
Number(n)	19	32	43
Age(year)	30.053 ± 7.40	38.375 ± 10.60	41.535 ± 8.56
Gender(male/female)	11/8	16/16	27/16
HBsAg (log10 IU/mL)	UD	2.654 ± 1.12	2.540 ± 1.05
HBV DNA (log10 IU/mL)	UD	3.374 ± 1.76	2.268 ± 0.63*
ALT(IU/L)	25.000 ± 5.47	37.994 ± 34.18	32.814 ± 13.17
AST(IU/L)	21.316 ± 4.35	27.656 ± 17.59	27.814 ± 8.3
WBC(×10^9^ /mL)	5.880 ± 0.65	5.569 ± 1.42	5.404 ± 1.50

HBsAg: hepatitis B surface antigen; ALT: alanine aminotransferase; AST: aspartate aminotransferase; WBC: white blood cells; The results are presented as the means ± SDs. *P < 0.05; untreated CHB vs. CHB treated with peg-IFN-α

UD: undetected

**Table 3:** Serological response during peg-IFN-α treatment

Serological response(n, %)	12w	24w	36w	48w
HBsAg clearance	1, 2.33%	4, 9.30%	5, 11.63%	11, 25.58%
HBsAg seroconversion	0, 0.0%	3, 6.98%	4, 9.30%	6, 13.95%

### TRIM19/38 mRNA levels in PBMCs of untreated CHB patients and healthy controls

**Fig. 1:**
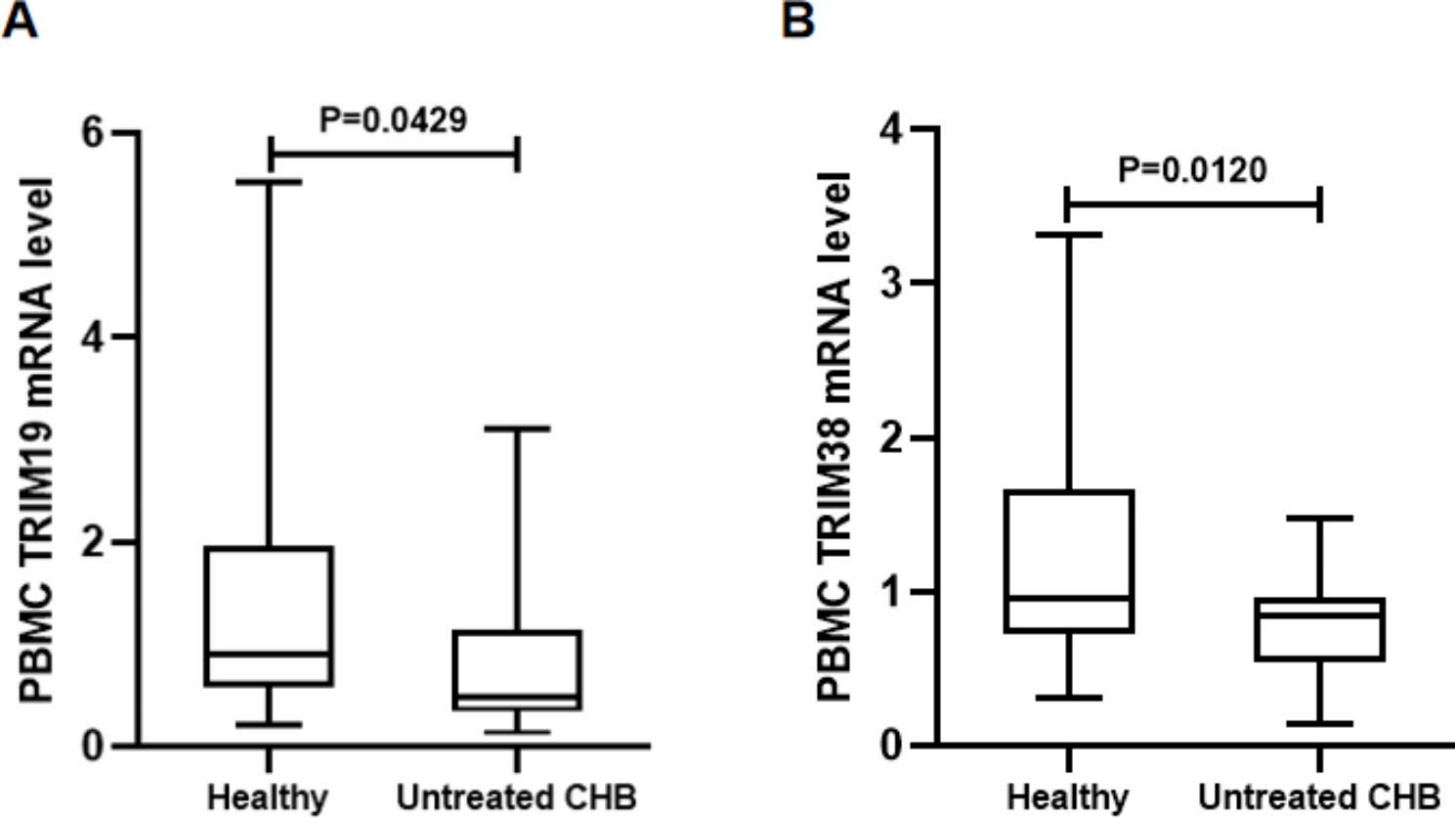
Comparison of TRIM19/38 mRNA levels in PBMCs from untreated CHB patients and healthy controls. **(A)** TRIM19 and **(B)** TRIM38 mRNA levels in PBMCs were analysed by qRT-PCR. The measurements were repeated three times. The expression level of TRIM19/38 was calculated by the 2^-ΔΔct method (Livak method) with β-actin as the reference gene

We first used qRT-PCR to detect TRIM19/38 mRNA levels in PBMCs from healthy controls and untreated CHB patients. Our results indicated that TRIM19 (P = 0.0429, Fig. 1A) and TRIM38 (P = 0.0120, Fig. 1B) mRNA levels in PBMCs of untreated CHB patients were significantly lower than those of healthy controls. These results suggest that TRIM19 and TRIM38 may be associated with HBV infection.

### Correlation of TRIM19/38 mRNA levels in PBMCs with virological indicators and ALT at baseline

**Fig. 2:**
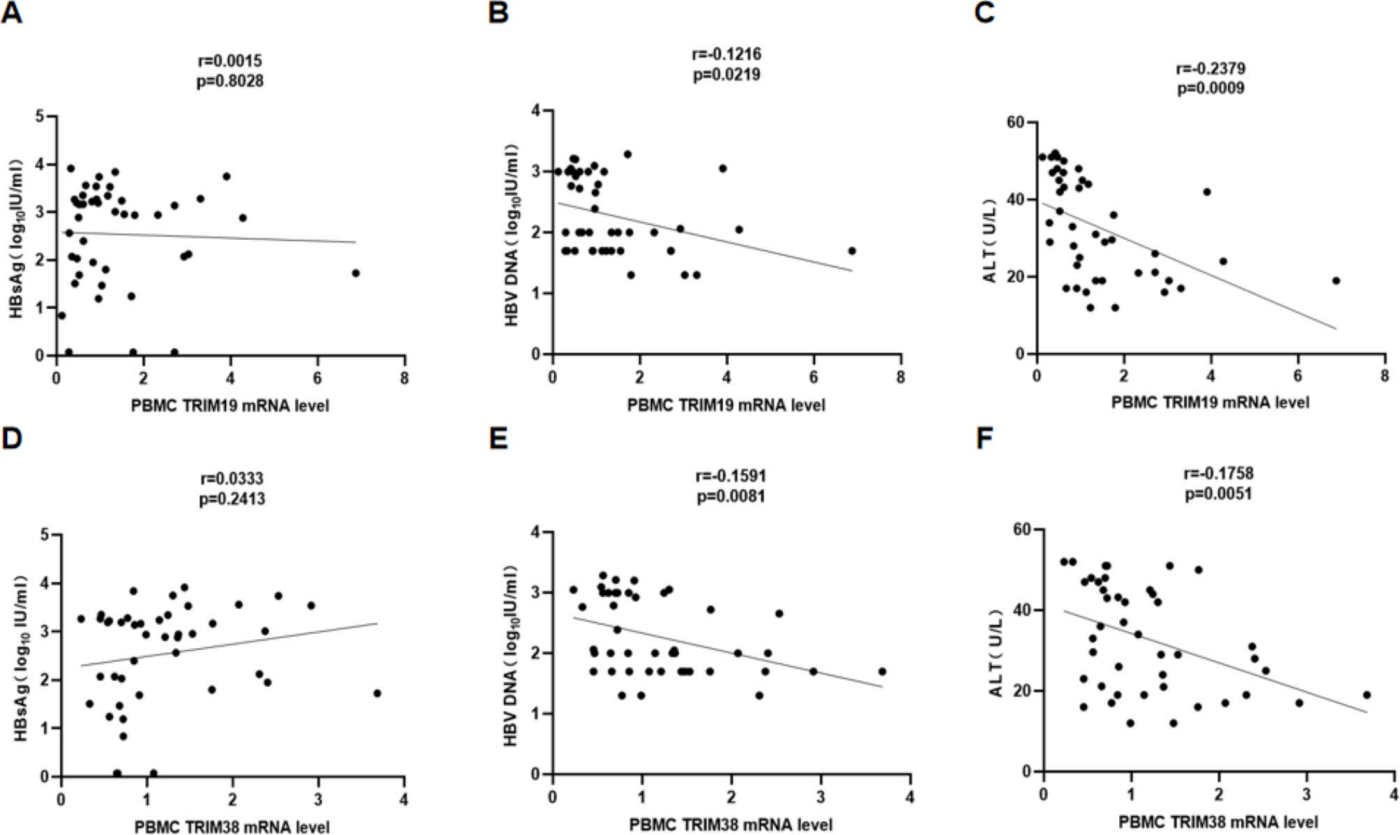
Correlations between TRIM19/38 mRNA levels and serological and virological indicators (HBsAg, HBV DNA and ALT) in HBeAg-negative CHB patients at baseline. Analysis of the correlation between TRIM19/38 mRNA levels and HBsAg (**A, D**), HBV DNA (**B, E**), and ALT (**C, F**) using Pearson’s test. TRIM19/38 mRNA levels in PBMCs were measured by qRT-PCR. The measurements were repeated three times. The expression level of TRIM19/38 was calculated by the 2^-ΔΔct method (Livak method) with β-actin as the reference gene. HBsAg and HBV DNA levels were log10 transformed. The correlation coefficient (r) and two-tailed p values were calculated via Pearson correlation. P < 0.05 was considered to be statistically significant

At baseline, we performed correlation analysis on TRIM19/38 mRNA levels in PBMCs of HBeAg-negative CHB patients and HBV virological characteristics. Correlation analysis revealed that there were no correlations between TRIM19/38 mRNA levels and HBsAg levels (Fig. 2A, D). Interestingly, a negative correlation between TRIM19 mRNA levels and HBV DNA (r=-0.1216, P = 0.0219) (Fig. 2B) and a negative correlation between TRIM38 mRNA levels and HBV DNA (r=-0.1591, P = 0.0081) (Fig. 2E) were observed. Consistent with this, TRIM19 mRNA levels were negatively correlated with ALT (r=-0.2379, P = 0.0009) (Fig. 2C) and TRIM38 mRNA levels were negatively correlated with ALT (r=-0.1758, P = 0.0051) (Fig. 2F).

### Dynamic changes in TRIM19/38 mRNA levels in PBMCs from CHB patients at early time points of peg-IFN-α therapy

In our study, 43 HBeAg (-) patients with CHB were treated with peg-IFN-α and followed up for 48 weeks. We divided CHB patients into a response (R) group and a nonresponse (NR) group based on the changes in HBV DNA and HBsAg levels at week 48 of peg-IFN-α therapy. Among them, 16 patients (9 males and 7 females) achieved peg-IFN-α response, and 27 (18 males and 9 females) did not. The baseline serological virological indicators of the two groups were compared. We found no significant difference in serum HBsAg between the two groups (Table 4), but the HBV DNA titers in the R group were lower than those in the NR group (P = 0.0022) as shown in Table 4. In contrast, the levels of ALT (P = 0.0025,Table 4) in the R group were higher than those in the NR group. As shown in Table 4, at 12 weeks and 24 weeks of therapy, HBsAg (P = 0.0004(12w), P < 0.0001(24w)) in the R group was significantly lower than those in the NR group, but transaminases ALT(P = 0.0019(12w)) and AST(P = 0.0001(12w)) in the R group were significantly higher than those in the NR group.

**Fig. 3:**
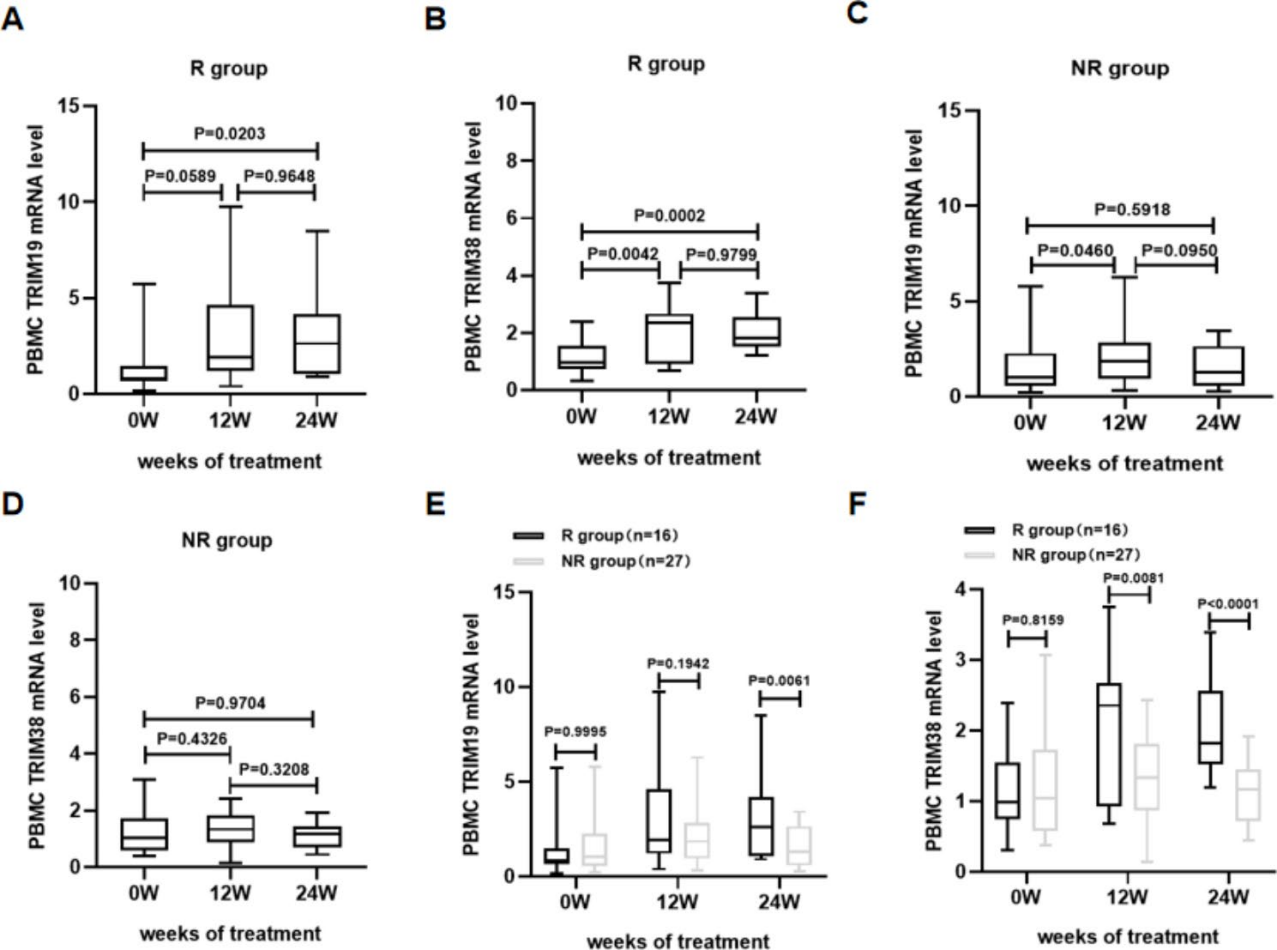
Transverse and longitudinal analysis of TRIM19/38 mRNA levels in PBMCs of CHB patients at early time points of peg-IFN-α therapy. (**A-B**) Dynamic changes in TRIM19/38 mRNA levels during therapy in the R group. (**C-D**) Dynamic changes in TRIM19/38 mRNA levels during therapy in the NR group. (**E-F**) TRIM19/38 mRNA levels in the R group and NR group were compared before therapy and at 12 weeks and 24 weeks of therapy. TRIM19/38 mRNA levels in PBMCs were measured by qRT-PCR. The measurements were repeated three times. The expression level of TRIM19/38 was calculated by the 2^-ΔΔct method (Livak method) with β-actin as the reference gene. P < 0.05 was statistically significant

To investigate the dynamic changes in TRIM19/38 mRNA levels in PBMCs during early antiviral therapy and their relationship with early therapy response, we detected and analysed TRIM19/38 mRNA levels in PBMCs of CHB patients in two groups (R and NR group) at the early stage of therapy (12 and 24 weeks). We found that TRIM19/38 mRNA levels were increased in the R and NR groups at the early stage of therapy (Fig. 3A-D). In the R group, the increase in TRIM19 mRNA levels was statistically significant at 24 weeks (P = 0.0203, Fig. 3A), and the increase in TRIM38 mRNA levels was statistically significant at both 12 (P = 0.0042, Fig. 3B) and 24 (P = 0.0002, Fig. 3B) weeks. In the NR group, TRIM19/38 mRNA levels were not significantly increased (Fig. 3C,D). We further compared TRIM19 and TRIM38 mRNA levels between the R and NR groups. We found that TRIM19/38 mRNA levels in the R group were higher than those in the NR group at weeks 12 and 24 of therapy (Fig. 3E,F). TRIM19 mRNA levels in the R group were significantly higher than those in the NR group at 24 weeks (P = 0.0061, Fig. 3E). TRIM38 mRNA levels in the R group were significantly higher than those in the NR group at both 12 (P = 0.0081, Fig. 3F) and 24 (P < 0.0001, Fig. 3F) weeks. Collectively, these results indicate the different expression dynamics of TRIM19/38 mRNA levels in the R and NR groups during early therapy with peg-IFN-α, and higher TRIM19/38 mRNA levels were associated with a better response to peg-IFN-α therapy.

**Table 4:** Comparison of clinical characteristics between peg-IFN-α response (R) group and nonresponse (NR) group

Characteristics	All (n = 43)	Baseline		P value	12w		P value	24w		P value
		R group (n = 16)	NR group (n = 27)		R group (n = 16)	NR group (n = 27)		R group (n = 16)	NR group (n = 27)	
Gender(male/female)	27/16	9/7	18/9		9/7	18/9		9/7	18/9	
Age(year)	41.535 ± 8.56	41.875 ± 8.90	41.370 ± 8.45	0.8537	41.875 ± 8.90	41.370 ± 8.45	0.8537	41.875 ± 8.90	41.370 ± 8.45	0.8537
HBsAg(log10 IU/mL)	2.540 ± 1.05	2.242 ± 0.86	2.717 ± 1.13	0.1533	1.153 ± 1.61	2.708 ± 1.02	0.0004	0.160 ± 1.87	2.599 ± 1.05	< 0.0001
HBV DNA (log10 IU/mL)	2.268 ± 0.63*	2.635 ± 0.57	3.186 ± 1.16	0.0022	1.773 ± 0.24*	1.824 ± 0.59****	0.7038	1.673 ± 0.18*	1.867 ± 0.51****	0.0621
ALT(IU/L)	32.814 ± 13.17	40.425 ± 11.61	28.304 ± 12.07	0.0025	78.688 ± 48.90	43.630 ± 19.75	0.0019	76.938 ± 82.83	49.074 ± 31.76	0.1233
AST(IU/L)	27.814 ± 8.30	32.144 ± 7.73	25.248 ± 7.65	0.2614	63.375 ± 26.02	37.852 ± 14.03	0.0001	64.938 ± 49.96	46.926 ± 29.80	0.1450
WBC(×10^9^ /mL)	5.404 ± 1.50	5.449 ± 1.63	5.378 ± 1.46	0.8832	3.079 ± 1.00	3.580 ± 1.11	0.1465	3.059 ± 0.93	3.528 ± 1.10	0.1603

The results were measured as the mean ± SDs and statistically analysed. *P < 0.05, ****P < 0.0001

### Changes in TRIM19/38 mRNA levels at week 24 of treatment could predict the therapeutic efficacy of peg-IFN-α treatment

**Fig. 4:**
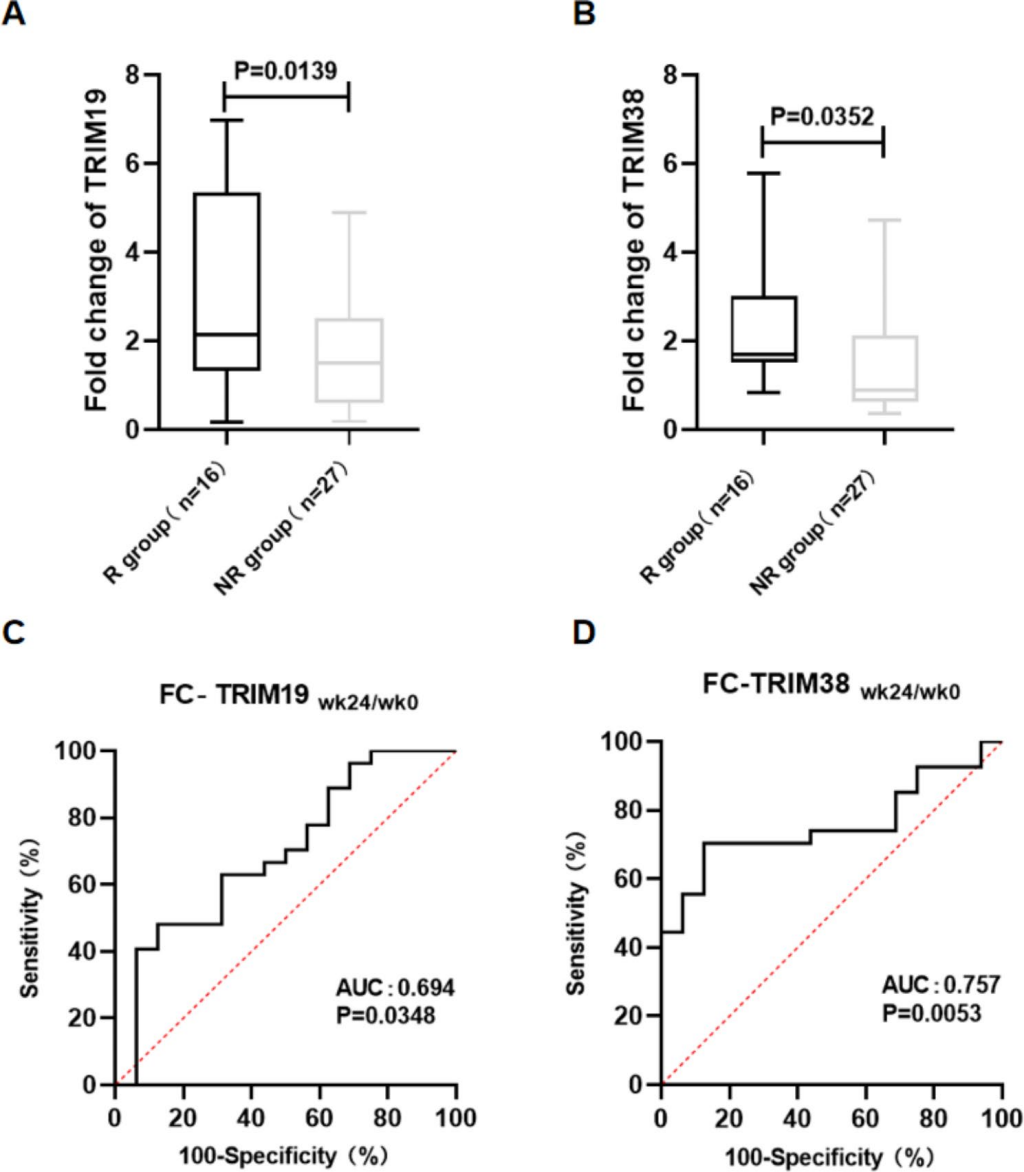
The change in TRIM19/38 at week 24 of peg-IFN-α treatment is associated with the response to treatment. (**A-B**) Fold change in TRIM19/38 mRNA levels from baseline to 24 weeks of treatment in PBMCs of the R and NR groups. FC-TRIM19/38_wk24/wk0_ was calculated as described in the Materials and Methods section. (**C-D**) Areas under the receiver operating characteristic curve for FC-TRIM19/38_wk24/wk0_. AUC: Areas under the receiver operating characteristics curves

Next, we evaluated the relationship between TRIM19/38 mRNA levels in PBMCs of CHB patients and the efficacy of peg-IFN-α treatment. We found that the change in TRIM19 mRNA levels from baseline to 24 weeks of treatment (FC-TRIM19 _(wk24/wk0)_) in PBMCs of the R group was significantly higher than that of the NR group (P = 0.0139, Fig. 4A). Similarly, FC-TRIM38_(wk24/wk0)_ in the R group was significantly higher than that in the NR group (P = 0.0352, Fig. 4B). Next, the receiver operating characteristic (ROC) curve analysis was used to evaluate the value of FC-TRIM19/38_(wk24/wk0)_ in predicting treatment response to peg-IFN-α therapy in CHB patients. The area under the curve (AUC) was calculated to obtain the best cut-off value of FC-TRIM19/38_(wk24/wk0)_. For treatment responders, the AUC of FC-TRIM19_(wk24/wk0)_ was 0.694 (P = 0.0348, Fig. 4C) and the AUC of FC-TRIM38_(wk24/wk0)_ was 0.757 (P = 0.0053, Fig. 4D). Regarding the FC-TRIM19_(wk24/wk0)_, the best cut-off value to predict treatment response was 1.263, and the sensitivity and specificity were 48.15% and 87.50%, respectively. Regarding the FC-TRIM38_(wk24/wk0)_, the best cut-off value to predict treatment response was 1.381, and the sensitivity and specificity were 70.37% and 87.50%, respectively. These data indicate that changes in TRIM19/38 mRNA levels at week 24 of treatment correlate with treatment response and are suitable for predicting the therapeutic effect of peg-IFN-α treatment.

### TRIM19/38 mRNA levels in PBMCs from CHB patients and serological response

To investigate whether TRIM19/38 mRNA levels in PBMCs are associated with serological response in CHB patients, we divided CHB patients treated with peg-IFN-α into a serological response (SR) group and a nonserological response (NSR) group based on whether serum HBsAg was lost or converted. After 48 weeks of peg-IFN-α therapy, 11 patients (6 males and 5 females) achieved a serological response and 32 (21 males and 11 females) did not. A comparison of the clinical characteristics of early therapy between the two groups is shown in Table 5. We found no significant differences in baseline HBV DNA titers, ALT, and AST between the two groups, but serum HBsAg was lower in the SR group than in the NSR group (P = 0.0006). As shown in Table 5, serum HBsAg (P < 0.0001 (12w), P < 0.0001 (24w)) in the SR group was significantly lower than that in the NSR group, but ALT (P < 0.0001 (12w)) and AST (P = 0.0001 (12w)) in the SR group were significantly higher than that in the NSR group.

**Fig. 5:**
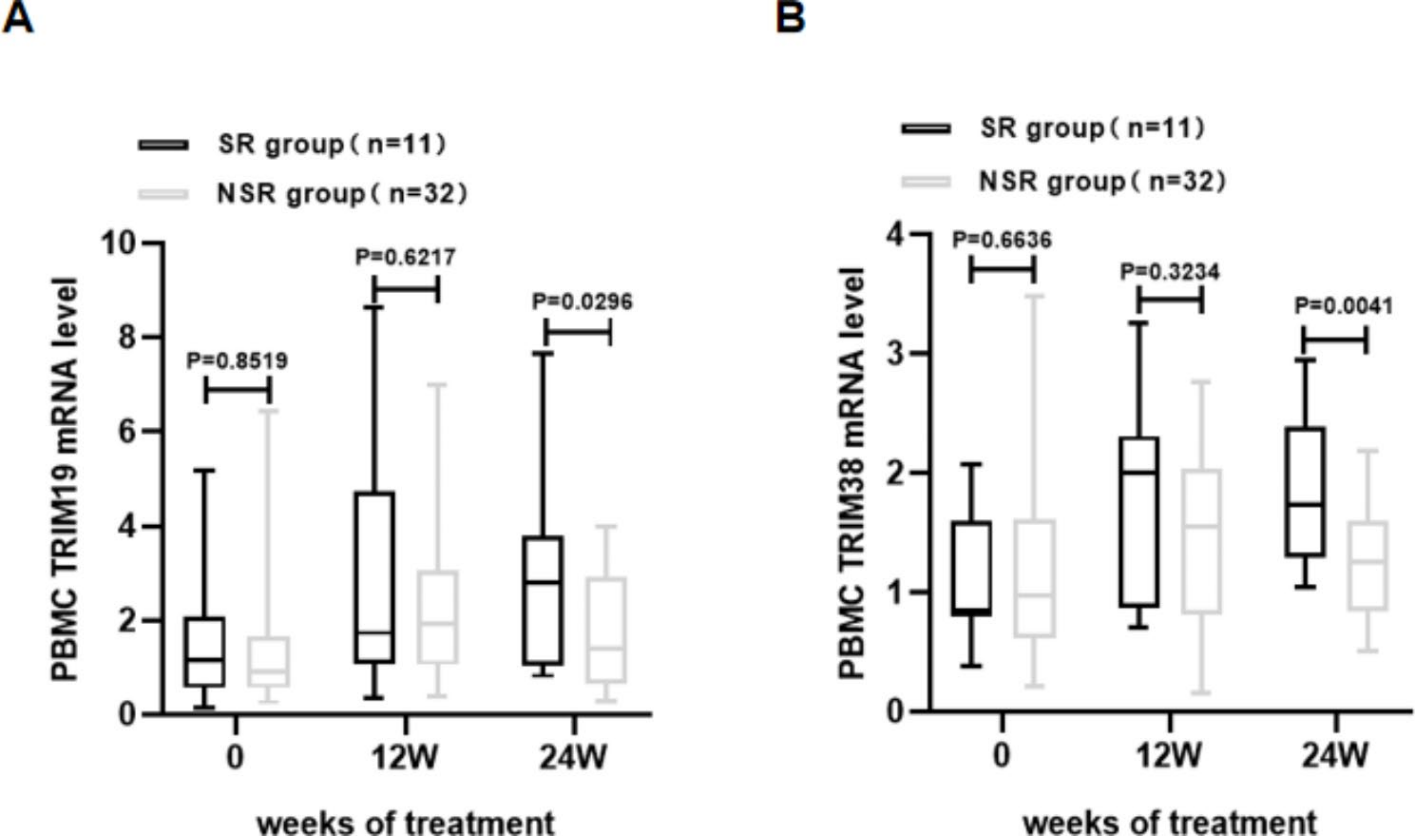
TRIM19/38 mRNA levels in the SR and NSR groups were compared at the early stage of therapy. (**A**) TRIM19 and (**B**) TRIM38 mRNA levels in the SR group and NSR group were compared before therapy and at 12 weeks and 24 weeks of therapy. TRIM19/38 mRNA levels in PBMCs were measured by qRT-PCR. The measurements were repeated three times. The expression level of TRIM19/38 was calculated by the 2^-ΔΔct method (Livak method) with β-actin as the reference gene. P < 0.05 was statistically significant

TRIM19/38 mRNA levels were detected and analysed in the two groups of CHB patients (SR and NSR groups) at the early stage of treatment (12 and 24 weeks). We found that TRIM19/38 mRNA levels in the SR and NSR groups were increased at the early stage of treatment, but there was no statistical significance. We further compared TRIM19/38 mRNA levels between the SR and NSR groups. We found that TRIM19 (P = 0.0296, Fig. 5A) and TRIM38 (P = 0.0041, Fig. 5B) mRNA levels in the SR group were higher than those in the NSR group at week 24 of therapy. The above results suggest that higher TRIM19/38 mRNA levels may be associated with the serological response to HBsAg during early peg-IFN-α treatment.

**Table 5:** Comparison of clinical characteristics between the serological response (SR) group and the nonserological response (NSR) group

Characteristics	All (n = 43)	Baseline		P value	12w		P value	24w		P value
		SR group (n = 11)	NSR group (n = 32)		SR group (n = 11)	NSR group (n = 32)		SR group (n = 11)	NSR group (n = 32)	
Gender(male/female)	27/16	6/5	21/11		6/5	21/11		6/5	21/11	
Age(year)	41.535 ± 8.56	39.818 ± 9.21	42.150 ± 1.05	0.4388	39.818 ± 9.21	42.150 ± 1.05	0.4388	39.818 ± 9.21	42.150 ± 1.05	0.4388
HBsAg(log10 IU/mL)	2.540 ± 1.05	1.863 ± 0.74	2.773 ± 1.05*	0.0006	0.421 ± 1.39	2.717 ± 0.95	< 0.0001	-0.819 ± 1.01	2.586 ± 0.96	< 0.0001
HBV DNA (log10 IU/mL)	2.268 ± 0.63*	2.170 ± 0.61	2.554 ± 0.61	0.0811	1.690 ± 0.16*	1.845 ± 0.55**	0.3001	1.663 ± 0.12**	1.840 ± 0.48***	0.2269
ALT(IU/L)	32.814 ± 13.17	37.691 ± 13.03	31.138 ± 13.00	0.1570	91.909 ± 53.28	44.563 ± 19.37	< 0.0001	82.000 ± 92.37	51.688 ± 37.71	0.1304
AST(IU/L)	27.814 ± 8.30	29.209 ± 7.21	27.334 ± 8.70	0.5249	68.636 ± 27.33	40.031 ± 15.71	0.0001	60.455 ± 52.43	51.281 ± 33.90	0.5073
WBC(×10^9^ /mL)	5.404 ± 1.50	5.645 ± 1.66	5.322 ± 1.46	0.5452	3.052 ± 1.85	3.511 ± 1.15	0.2319	3.220 ± 0.93	3.399 ± 1.10	0.6311

The results were measured as the mean ± SDs and statistically analyzed. *P < 0.05, **P < 0.01,***P < 0.001.

## Discussion

Although peg-IFN-α-based therapy can help HBeAg-negative chronic HBV carriers achieve faster and better HBV suppression and possibly a clinical cure, thereby reducing the occurrence of end-stage liver disease [20], there are still some patients with a negative response, so the efficacy of peg-IFN-α therapy still needs to be evaluated and optimized. In this study, we found that TRIM19/38 mRNA levels in PBMCs during peg-IFN-α treatment were not only predictive of early peg-IFN-α treatment efficacy, but also associated with HBsAg loss in HBeAg-negative chronic HBV carriers. We obtained the following results:(1) The levels of TRIM19/38 mRNA in untreated CHB patients were lower than those in normal controls; (2) At baseline, TRIM19/38 mRNA levels were negatively correlated with HBV DNA and ALT in HBeAg-negative chronic HBV carriers; (3) At the early stage of peg-IFN-α treatment, the dynamic changes in TRIM19/38 mRNA levels in PBMCs of R and NR groups were different, and higher levels of TRIM19/38 mRNA were associated with peg-IFN-α treatment; (4) The change in TRIM19/38 mRNA levels at week 24 of treatment could predict the therapeutic effect of peg-IFN-α treatment; and (5) Higher TRIM19/38 mRNA levels may be associated with HBsAg loss during early peg-IFN-α treatment.

One study pointed out that it would be of interest to analyse genes associated with disease prediction in PBMCs of patients [21]. Lu et al. found that interferon gamma-inducible protein 16 (IFI16) in peripheral blood mononuclear cells of CHB patients sensed hepatitis B virus infection and regulated antiviral immunity [22]. Recent studies have also noted that Toll-like receptor 8 (TLR8) in PBMCs of CHB patients is not only related to HBV infection, but can also predict the therapeutic efficacy of peg-IFN-α [23]. Similarly, TRIM25, a triple motif family protein, is downregulated in PBMCs of CHB patients and is associated with HBV infection [24]. Previous studies reported that TRIM19 [19] and TRIM38 [15] can inhibit HBV and can be induced by IFN-α. Therefore, we first examined the levels of TRIM19/38 mRNA in PBMCs of healthy controls from untreated CHB patients, and the results showed that the levels of TRIM19/38 mRNA in untreated CHB patients were lower than those in normal controls. Interestingly, TRIM19/38 mRNA levels were negatively correlated with HBV DNA and ALT in HBeAg-negative chronic HBV carriers at baseline. This may be due to the inhibition of TRIM19/TRIM38 by HBV and proves that TRIM19/38 may be related to HBV infection.

Current treatment regimens can control the development of CHB, but the efficacy and response still need to be optimized, so the prediction of the efficacy of peg-IFN-α is particularly important. Therefore, we hypothesized that TRIM19/38 mRNA levels in PBMCs of HBeAg-negative chronic HBV carriers would be associated with the efficacy of peg-IFN-α treatment. As expected, our findings showed that the dynamic changes in TRIM19/38 mRNA levels in PBMCs of the R and NR groups showed different patterns during early peg-IFN-α treatment. TRIM19/38 mRNA levels were significantly higher in the R group than in the NR group, especially at 24 weeks of treatment. Interestingly, we evaluated the predictive value of changes in TRIM19/38 mRNA levels during early treatment for the efficacy of peg-IFN-α treatment in CHB patients by ROC curve analysis. Changes in the mRNA levels of TRIM19 and TRIM38 predicted treatment response, with AUCs of 0.694 and 0.757, respectively. Our results suggest that TRIM19/38 mRNA levels in PBMCs of HBeAg-negative chronic HBV carriers during early treatment with peg-IFN-α may be associated with better therapeutic efficacy and prognosis. Therefore, it can provide good help for clinicians to judge whether to continue peg-IFN-α treatment to make better use of medical resources and better improve the treatment effect of CHB patients. Therefore, higher TRIM19/38 mRNA levels in PBMCs of CHB patients may serve as a useful biomarker to predict response to peg-IFN-α treatment during early treatment.

“Clinical cure” was defined as HBsAg clearance with or without the presence of anti-HBs [25]. Currently, “clinical cure” is considered the ultimate goal of antiviral therapy in CHB patients and is associated with favorable long-term clinical outcomes [26, 27]. However, only a small proportion of patients achieve this end point. A recent study showed that low HBsAg levels at baseline were predictive of HBsAg clearance in HBeAg-negative CHB patients treated with peg-IFN-α [28]. This is consistent with our study, in which patients who achieved HBsAg clearance had significantly lower baseline HBsAg levels than those who did not. Interestingly, by analysing the levels of TRIM19/38 mRNA in PBMCs of CHB patients in the SR and NSR groups during early treatment with peg-IFN-α, we found that TRIM19/38 mRNA levels were significantly higher in the SR group than the NSR group at 24 weeks of treatment. Collectively, TRIM19/38 mRNA levels are associated with HBsAg clearance in HBeAg-negative CHB patients and may serve as a biomarker to predict “clinical cure” in CHB patients.

In conclusion, our data suggest that higher TRIM19/38 mRNA levels in PBMCs of HBeAg-negative CHB patients may be associated with early treatment efficacy and HBsAg clearance with peg-IFN-α. This may suggest that TRIM19/38 has the potential to be a useful biomarker to help clinical treatment achieve a better response, reduce unnecessary side effects in nonresponders, and provide support for controlling CHB development and achieving a “clinical cure”. Further data is required to prove this.

## Data Availability

The data that support the findings of this study are available from the corresponding author upon reasonable request.
